# Oleoylethanolamide Ameliorates Dextran Sulfate Sodium-Induced Colitis in Rats

**DOI:** 10.3389/fphar.2020.01277

**Published:** 2020-08-14

**Authors:** Shinsuke Otagiri, Shunsuke Ohnishi, Masatsugu Ohara, Qingjie Fu, Koji Yamamoto, Keiko Yamamoto, Takehiko Katsurada, Naoya Sakamoto

**Affiliations:** Department of Gastroenterology and Hepatology, Hokkaido University Graduate School of Medicine, Sapporo, Japan

**Keywords:** oleoylethanolamide, dextran sulfate sodium, inflammatory bowel disease, nuclear factor kappa B, peroxisome proliferator-activated receptor-α

## Abstract

Oleoylethanolamide (OEA) is an endogenous fatty acid ethanolamide known for its anti-inflammatory effects and its influence on gut microbiota composition; however, the effects of OEA in inflammatory bowel disease (IBD) remain unknown. During *in vitro* experiments, OEA downregulated the expression of tumor necrosis factor (TNF)-α and reduced phosphorylation of inhibitor of kappa (Iκ) Bα induced by lipopolysaccharide in human embryonic kidney cells. Moreover, OEA downregulated the expression of interleukin (IL)-8 and IL-1β and inhibited the phosphorylation of IκBα and p65 induced by TNF-α in human enterocytes (Caco-2). The effect of OEA in reducing the expression of IL-8 was blocked by the peroxisome proliferator-activated receptor (PPAR)-α antagonist. During *in vivo* experiments on rats, colitis was induced by the oral administration of 8% dextran sulfate sodium from day 0 through day 5, and OEA (20 mg/kg) was intraperitoneally injected once a day from day 0 for 6 days. OEA administration significantly ameliorated the reduction in body weight, the increase in disease activity index score, and the shortening of colon length. In rectums, OEA administration reduced the infiltration of macrophages and neutrophils and tended to reduce the histological score and the expression of inflammatory cytokines. Administration of OEA produced significant improvement in a colitis model, possibly by inhibiting the nuclear factor kappa B signaling pathway through PPAR-α receptors. OEA could be a potential new treatment for IBD.

## Introduction

Inflammatory bowel disease (IBD), which mainly consists of Crohn’s disease (CD) and ulcerative colitis (UC), is a chronic inflammatory disorder of the gastrointestinal tract. Due to the development of new therapeutic drugs for IBD in recent decades, such as biological medicines, treatment options have increased (Feagan et al., 2016; Sandborn et al., 2017); however, due to a lack of curative treatment for IBD, many patients remain resistant to medical therapy and require surgery (Peyrin-Biroulet et al., 2010; Fumery et al., 2018). Therefore, new therapeutic options for IBD are needed.

Oleoylethanolamide (OEA) is an endogenous fatty acid ethanolamide belonging to the N-acylethanolamine family. OEA is contained in foods such as nuts, cocoa powder, and oatmeal (Payahoo et al., 2019), and has been found in different tissues such as the gastrointestinal tract, muscle, adipocytes, liver, kidney, heart, lung, pancreas, brain, salivary gland, and reproductive tract (Ambrosini et al., 2006; Tutunchi et al., 2020). OEA activates peroxisome proliferator-activated receptor (PPAR)-α, G protein-coupled receptor (GPR) 119, and transient receptor potential vanilloid (TRPV) 1 (Brown et al., 2016). Previous studies have demonstrated that the administration of OEA has therapeutic effects on modulating feeding, lipid metabolism, and gastrointestinal motility (Cluny et al., 2009; Decara et al., 2012; Fu and Gaetani, 2013). Other studies have demonstrated that OEA has neuroprotective and anti-atherosclerotic functions (Fan et al., 2014; Gonzalez-Aparicio et al., 2017). OEA has also been receiving attention for its anti-inflammatory effects and its effect on gut microbiota composition (Di Paola et al., 2018; Yang et al., 2016). A study on humans has shown that plasma OEA levels are elevated in patients with CD, and that these levels correlate with disease severity (Grill et al., 2019), suggesting a role for OEA in reinstating homeostasis. However, the effects and roles of OEA administration in IBD remain unknown.

Thus, the aim of this study was to examine the effects of OEA on colitis in rats and to investigate their underlying mechanisms.

## Materials and Methods

### Reagents

OEA and MK866, a PPAR-α antagonist, were purchased from Cayman Chemical (Ann Arbor, MI, USA). OEA was dissolved in dimethyl sulfoxide (DMSO, Wako Pure Chemical Industries, Osaka, Japan) for the *in vitro* experiments and was dissolved in a vehicle composed of Tween 80 (Kanto Chemical, Tokyo, Japan), DMSO, and phosphate-buffered saline (PBS, Life Technologies, Carlsbad, CA, USA) (1:0.5:18.5 by volume) for the *in vivo* experiments.

### Cell Culture

Caco-2 cells (human intestinal epithelial cells) were purchased from RIKEN BioResource Center (Tsukuba, Japan). The cells were cultured in Dulbecco’s modified Eagle’s medium (DMEM, Thermo Fisher Scientific, Waltham, MA, USA) containing 10% fetal bovine serum (FBS, Moregate Biotech, Bulimba, Australia), 100 U/ml penicillin, and 100 μg/ml streptomycin (Wako Pure Chemical Industries); and 1% MEM non-essential amino acids solution (Wako Pure Chemical Industries), and was maintained at 37°C in a humidified atmosphere of 5% CO_2_. The medium was changed every other day. Caco-2 cells were activated by human recombinant tumor necrosis factor (TNF)-α (Merck Millipore, Burlington, MA, USA). Human embryonic kidney cells (HEK293, RIKEN BioResource Center) or their derivatives, which were stably transfected with the human toll-like receptor (TLR) 4a, MD2, and CD14 genes (293/hTLR4A-MD2-CD14; InvivoGen, San Diego, CA, USA), were cultured in DMEM containing 10% FBS, 100 U/ml penicillin, and 100 μg/ml streptomycin. The 293/hTLR4A-MD2-CD14 cells were activated by lipopolysaccharide (LPS, Sigma-Aldrich, St. Louis, MO, USA).

### Western Blot Analysis

To investigate phosphorylation of the inhibitor of kappa (Iκ) Bα and p65, Caco-2 cells and 293/hTLR4A-MD2-CD14 cells were plated into 6-well plates (2 × 10^5^ cells/well; Corning, NY, USA) and cultured. After the cells became nearly confluent, the culture medium was changed to a medium containing OEA (40 μM) or DMSO, and the cells were incubated for an additional 30 min. The cells were then treated with 5.0 ng/ml TNF-α for 15 min or 10 ng/ml LPS for 2 h and were washed with ice-cold PBS. Cell lysates were prepared using a radioimmunoprecipitation assay buffer containing 50 mM Tris-HCl (pH 8.0), 150 mM NaCl, 0.5% (w/v) sodium deoxycholate, 0.1% (w/v) sodium dodecyl sulfate (SDS), 1.0% (w/v) NP-40 substitute, and Protease/Phosphatase Inhibitor Cocktail (Cell Signaling Technology, Beverly, MA, USA). Equal amounts of cellular protein extracts were diluted in a 4 × Laemmli sample buffer (Bio-Rad, Hercules, CA, USA). The samples were heated at 95°C for 5 min, then underwent SDS-polyacrylamide gel electrophoresis (Bio-Rad). The separated proteins were transferred to Immobilon-P polyvinylidene difluoride membranes (Merck Millipore), which were subsequently incubated in tris-buffered saline with 0.05% Tween 20 (Wako Pure Chemical industries) consisting of a 5% PhosphoBLOCKER blocking reagent (Cell Biolabs, San Diego, CA, USA) at room temperature for 60 min. The membranes were probed with primary antibodies for phospho-IκBα (1:2,000; Cell Signaling Technology), IκBα (1:2,000; Cell Signaling Technology), phospho-p65 (1:2,000; Cell Signaling Technology), and nuclear factor kappa B (NF-κB, 1:2,000; Cell Signaling Technology); actin (1:2,000; Abcam, Cambridge, UK) and bound antibodies were detected with peroxidase AffiniPure Goat Anti-Mouse IgG (H+L) (1:10,000; Jackson ImmunoResearch, West Grove, PA, USA) or peroxidase AffiniPure Goat Anti-Rabbit IgG (H+L) (1:10,000; Jackson ImmunoResearch), and visualized and photographed using ECL Prime detection reagent (GE Healthcare, Chicago, IL, USA). The blots were analyzed using Fusion Solo S (Vilber Lourmat, Marne-la-Vallée, France).

### Animals

Eight-week-old male Sprague-Dawley rats were procured from Japan SLC (Hamamatsu, Japan), and one rat was housed per cage in a temperature-controlled room (24°C) on a 12 h light/12 h dark cycle. All rats had *ad libitum* access to standard pellets. We measured body weight, scored fresh feces for consistency, and observed bleeding each day to calculate the disease activity index (DAI) as described in **Table 1** (Pandurangan et al., 2015).

**Table 1:** Assessment of DAI. (Pandurangan et al., 2015).

Score	Weight loss	Stool consistency	Bleeding
0	No loss	Normal	No blood
1	1–5%		
2	5–10%	Loose stool	Visual pellet bleeding
3	10–20%		
4	> 20%	Diarrhea	Gross bleeding/Blood around anus

### Induction of Colitis and OEA Treatment

**Figure 1:**
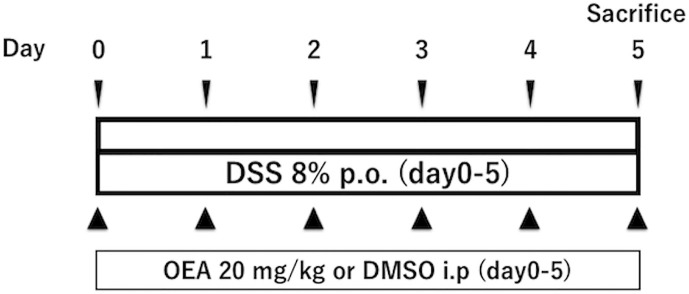
Experimental protocol for DSS-induced colitis. Rats received oral administration of 8% of DSS from day 0 through day 5. OEA (20 mg/kg) was intraperitoneally injected once a day from day 0 for 6 days. All rats were euthanized at day 5.

Colitis in the DSS group and the DSS + OEA group was induced by oral administration of 8% dextran sulfate sodium (DSS, M.W. = 36,000 - 50,000; MP Biomedicals, Solon, OH, USA) through drinking water from day 0 through day 5 (**Figure 1**) (Martin et al., 2016). The control group and the OEA group rats had *ad libitum* access to water. OEA (20 mg/kg) was intraperitoneally injected once a day from day 0 for 6 days to the OEA group and the DSS + OEA group. The control group and the DSS group were treated with DMSO. The dose of OEA was based on previously reported studies (Gaetani et al., 2003; Proulx et al., 2005).

### Histological Examination

The rats were euthanized after 6 days of DSS and OEA treatment. The colon was excised and we cut a fragment of distal colon for about 3 cm and carefully opened it. Then, we longitudinally cut the 3 cm fragment for 2 mm in width for quantitative reverse transcription-polymerase chain reaction (qRT-PCR). The rest of the fragment was used for histological examinations. The fragment was fixed in 40 g/L of formaldehyde saline, embedded in paraffin, and cut into 5 μm sections. Tissue sections were stained with hematoxylin and eosin (HE). Parameters of inflammation severity, inflammation extent, crypt damage, and percent involvement (percentage of damage area in the fragment) were scored in a blinded fashion. Then, the sum of these three scores (inflammation severity, inflammation extent, crypt damage) was multiplied by percent involvement (0%; ×0, 1–25%; ×1, 26–50%; ×2, 51–75%; ×3, 75–100%; ×4) as described in **Table 2** (Singh et al., 2011; Onishi et al., 2015).

**Table 2:** Colitis histology score. (Singh et al., 2011; Onishi et al., 2015).

Changes	Score	Degree
Inflammation severity	0	None
	1	Mild
	2	Moderate
	3	Severe
Inflammation extent	0	None
	1	Mucosa
	2	Mucosa and submucosa
	3	Transmural
Crypt damage	0	None
	1	Basal 1/3 damaged
	2	Basal 2/3 damaged
	3	Crypts lost; surface epithelium present
	4	Crypts and surface epithelium lost
Percent involvement	0	0%
	1	1–25%
	2	26–50%
	3	51–75%
	4	75–100%

### Immunohistochemical Examination

To assess macrophage infiltration, the tissue sections were stained with anti-rat CD68 monoclonal antibody (1:100; AbD Serotec, Kidlington, UK) for 60 min at room temperature. To assess neutrophil infiltration, the tissue sections were stained with anti-rat myeloperoxidase (MPO) antibody (1:300, Thermo Fisher Scientific) overnight at 4°C. We photographed 10 random fields on a section from each rat and measured the ratio of stained areas in the mucosal layer using a digital image analyzer (WinROOF, Mitani Co., Fukui, Japan).

### RNA Isolation and qRT-PCR

Total RNA of the cultured cells or the rat colon was extracted using the RNeasy Mini Kit (Qiagen, Hilden, Germany), and 1 μg of the total RNA was reverse transcribed into cDNA using the PrimeScript™ RT reagent Kit (Takara Bio, Kusatsu, Japan). PCR was performed using a 25 μl reaction mixture containing 1 μl of cDNA and 12.5 μl Platinum SYBR Green PCR Mix (Life Technologies). β-actin messenger RNA amplified from the same samples served as an internal control. After initial denaturation at 95°C for 2 min, we used a 2-step cycle procedure (denaturation at 95°C for 15 s, annealing and extension at 60°C for 1 min) for 40 cycles in a 7700 Sequence Detector (Applied Biosystems, Foster City, CA, USA). Gene expression levels were determined using the comparative threshold cycle (ΔΔCt) method with β-actin used as an endogenous control. Data were analyzed with Sequences Detection Systems software (Applied Biosystems). The primer sequences are shown in **Table 3**.

**Table 3:** Primer sequences used for qRT-PCR.

Gene	Forward primer (5’ to 3’)	Reverse primer (5’ to 3’)	Accession no.
human β-actin	ccaaccgcgagaagatga	ccagaggcgtacagggatag	XM_035025796.1
human IL-8	agagtgattgagagtggacc	acttctccacaaccctctg	XM_003832335.5
human IL-1β	aagctgatggccctaaacag	aggtgcatcgtgcacataag	XM_003804503.3
human TNF-α	cagcctcttctccttcctga	gccagagggctgattagaga	XM_003831589.2
rat β-actin	aagatgacccagatcatgtt	ttaatgtcacgcacgatttc	XM_017294214.2
rat IL-1β	cctatgtcttgcccgtggag	cacacactagcaggtcgtca	XM_032902343.1
rat TNF-α	accacgctcttctgtctactg	cttggtggtttgctacgac	XM_032888689.1
rat TGF-β	ctgctgacccccactgatac	agccctgtattccgtctcct	XM_034509875.1
rat IL-8	cattaatatttaacgatgtggatgcgtttca	gcctaccatctttaaactgcacaat	XM_032916528.1
rat PPAR-α	aatccacgaagcctacctga	gtcttctcagccatgcacaa	XM_034516616.1
rat CD68	tcacaaaaaggctgccactctt	tcgtagggcttgctgtgctt	NM_001031638.1

### Statistical Analysis

Data are expressed as mean ± standard deviation (SD). Parameters among the groups were compared by one-way analysis of variance, followed by the Holm-Sidak *post hoc* test. The difference was considered significant at *p* < 0.05. All analyses were performed using GraphPad Prism, version 7 (GraphPad software, San Diego, CA, USA).

## Results

### OEA Suppresses LPS-Induced Inflammatory Reaction *in Vitro*

**Figure 2:**
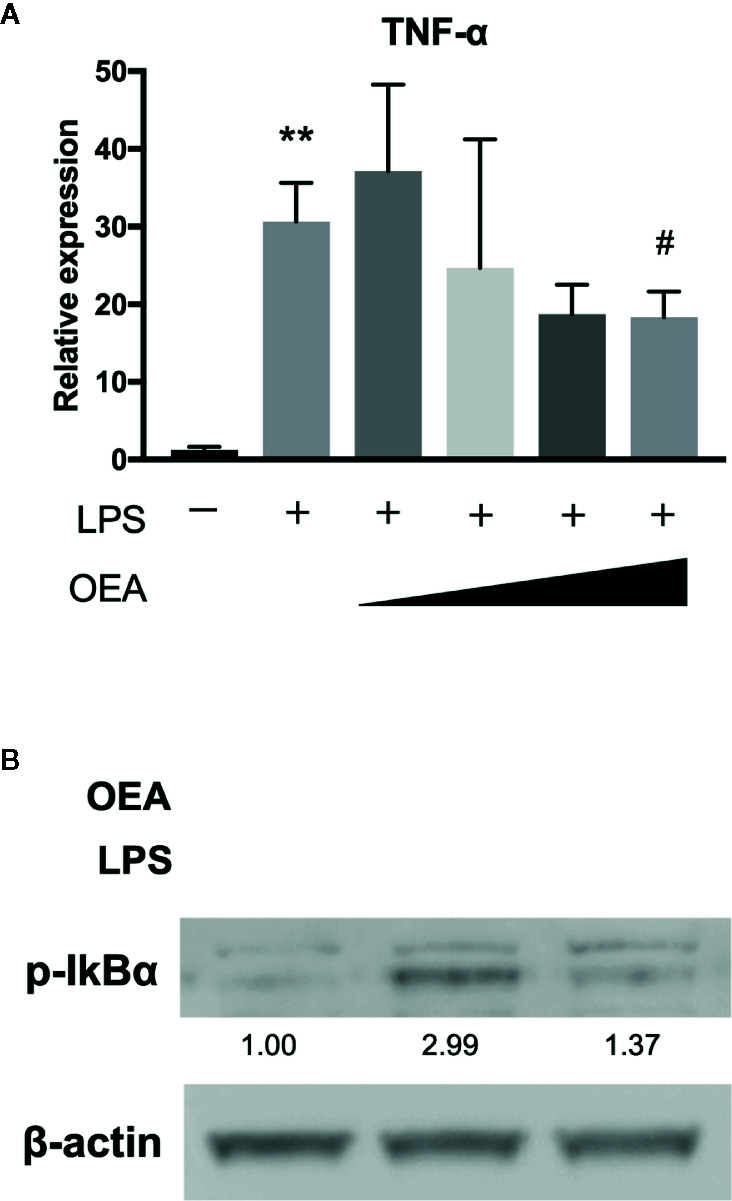
Effect of OEA on inflammatory reactions *in vitro*. **(A)** 293/hTLR4A-MD2-CD14 cells were treated with LPS (1 ng/ml) for 3 h after pretreatment with OEA (2, 5, 10, and 40 μM) for 30 min. Total RNA was isolated and the expression of TNF-α was investigated by qRT-PCR. **(B)** 293/hTLR4A-MD2-CD14 cells were treated with LPS (10 ng/ml) for 2 h after pretreatment with OEA (40 μM) for 30 min, and the expression of phospho-IκBα was investigated by Western blotting. Numeric data indicates relative average pixel intensity. The values are the mean ± SD (n = 3). **p < 0.01 versus the Control. ^#^ *p* < 0.05 versus LPS.

To investigate the effect of OEA on LPS-TLR4 signaling pathway, we first conducted *in vitro* experiments using 293/hTLR4A-MD2-CD14 cells, which stably express human TLR4A, MD2 and CD14. Treatment with LPS significantly upregulated the expression of TNF-α compared to control, whereas OEA significantly and dose-dependently reduced the expression of TNF-α compared to LPS treatment (**Figure 2A**). Western blotting demonstrated that treatment with LPS increased phosphorylation of IκBα compared to control, whereas OEA (40 μM) inhibited LPS-induced phosphorylation of IκBα in 293/hTLR4A-MD2-CD14 cells compared to LPS treatment (**Figure 2B**).

### OEA Suppresses TNF-α-Induced Inflammatory Reaction of Caco-2 Cells

**Figure 3:**
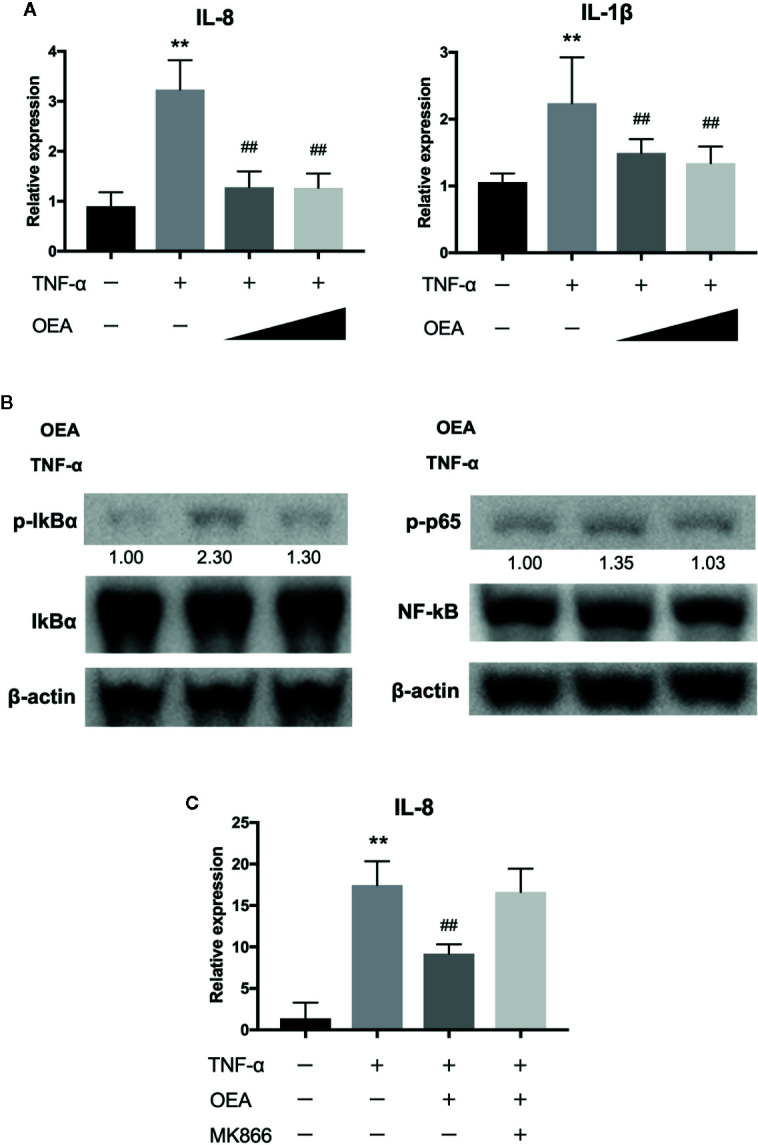
Effect of OEA on inflammatory reaction in Caco-2 cells. **(A)** Caco-2 cells were treated with TNF-α (5.0 ng/ml) for 1 h after pretreatment with OEA (10 and 40 μM) for 30 min. Total RNA was isolated and the expression of IL-8 and IL-1β were investigated by qRT-PCR. **(B)** Caco-2 cells were treated with TNF-α (5.0 ng/ml) for 15 min after pretreatment with OEA (40 μM) for 30 min, and the expression of phospho-IκBα and phospho-p65 was investigated by Western blotting. Numeric data indicates relative average pixel intensity. **(C)** Caco-2 cells were treated with TNF-α (5.0 ng/ml) for 1 h after pretreatment with OEA (40 μM) and MKK866 (10 μM) for 30 min. Total RNA was isolated and the expression of IL-8 was investigated by qRT-PCR. The values are the mean ± SD (n = 3). ^**^ *p* < 0.01 versus the Control. ^##^ *p* < 0.01 versus TNF-α.

We next investigated the anti-inflammatory effect of OEA on human intestinal epithelial cells (Caco-2 cells). Treatment with TNF-α significantly upregulated the expression of interleukin (IL)-8 and IL-1β compared to control, and OEA significantly reduced the expression of IL-8 and IL-1β compared to TNF-α treatment (**Figure 3A**). Western blotting demonstrated that treatment with TNF-α increased phosphorylation of IκBα and p65 compared to control, and OEA inhibited TNF-α-induced phosphorylation of IκBα and p65 in Caco-2 cells compared to TNF-α treatment (**Figure 3B**). These results suggest that OEA suppresses the activation of the NF-κB signaling pathway in intestinal epithelial cells. Moreover, the effect of OEA in reducing the expression of IL-8 was blocked by MKK866, a PPAR-α antagonist (**Figure 3C**).

### OEA Suppresses DSS-Induced Colitis in Rats

**Figure 4:**
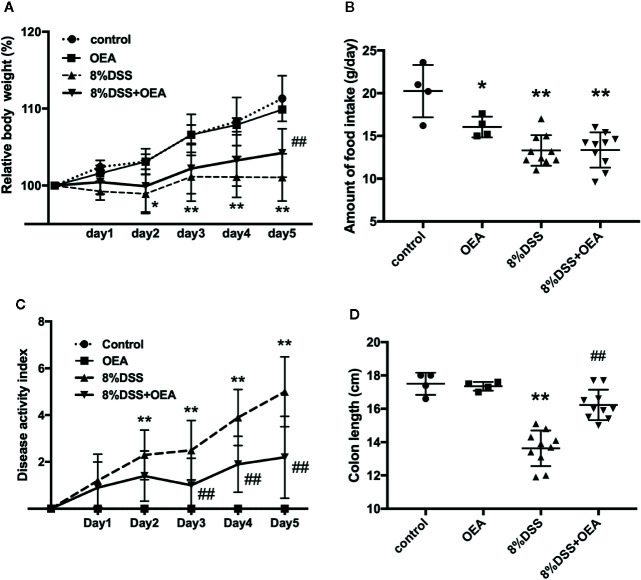
Effect of OEA on DSS-induced colitis in rats. **(A)** Relative body weight. **(B)** Amount of food intake. **(C)** DAI. **(D)** Colon length. The values are the mean ± SD (n = 4, Control group; n = 4, OEA group; n = 10, DSS group and n = 10, DSS + OEA group). ^*^ *p* < 0.05 versus Control group. ^**^ *p* < 0.01 versus Control group. ^##^ *p* < 0.01 versus DSS group.

We investigated the effects of OEA on DSS-induced colitis in rats. The weight gain in the DSS group was inhibited and the relative body weight in the DSS group was significantly less than that in the control group from day 2 onward (**Figure 4A**). However, this change was attenuated by OEA administration, and the relative body weight was significantly increased in the DSS + OEA group compared with the DSS group on day 5. Food intake was significantly reduced in the OEA group, the DSS group, and the DSS + OEA group compared with the control group (**Figure 4B**). There was no significant difference in food intake among the OEA group, the DSS group, and the DSS + OEA group. DAI gradually increased in the DSS group, but this increase was ameliorated by OEA administration; DAI in the DSS + OEA group was significantly lower than in the DSS group on day 3 onward (**Figure 4C**). Colon length in the DSS group was significantly shorter than in the control group, but the length was significantly longer in the DSS+OEA group compared with the DSS group (**Figure 4D**).

### Effect of OEA Administration on Histological Score and Infiltration of Inflammatory Cells

**Figure 5:**
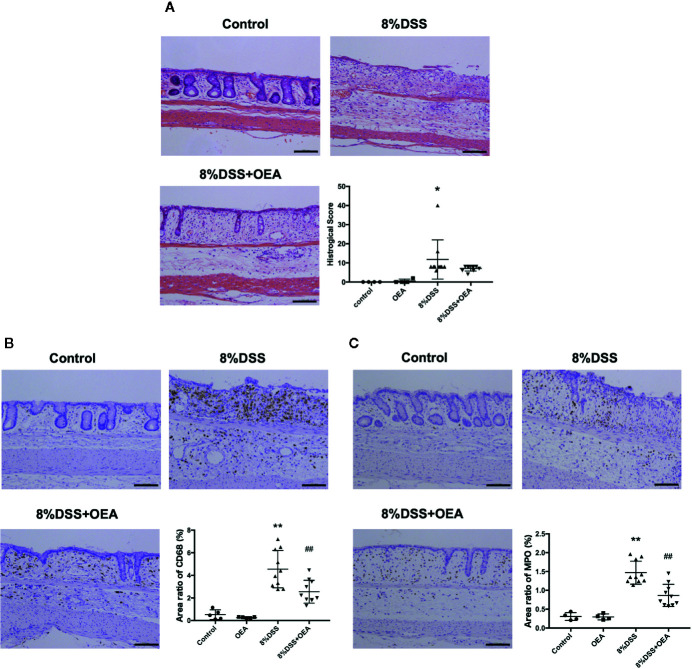
Histological analyses of the colon. **(A)** HE staining. **(B)** CD68 expression. **(C)** MPO expression. The stained areas were measured from the entire colon cross-sectional area. Scale bars, 100 μm. The values are the mean ± SD (n = 4, Control group: n = 4, OEA group; n = 10, DSS group and n = 10, DSS + OEA group). ^*^ *p <*0.05 versus Control group. ^**^ *p* < 0.01 versus Control group. ^##^ *p* < 0.01 versus DSS group.

On HE staining, severe transmural inflammation, loss of crypts, and infiltration of inflammatory cells were observed in the DSS group compared to the control group and these findings tended to be attenuated by the administration of OEA compared to the DSS group (**Figure 5A**). The histological score in the DSS group was significantly higher than in the control group, and the histological score in the DSS + OEA group tended to be lower than in the DSS group. The infiltration of CD68-positive macrophages was significantly increased in the DSS group compared to the control group; however, OEA administration significantly reduced the infiltration of macrophages compared to the DSS group (**Figure 5B**). The infiltration of MPO-positive neutrophils was significantly increased in the DSS group compared to the control group; however, OEA administration significantly suppressed this increase compared to the DSS group (**Figure 5C**).

### Effects of OEA Administration on Gene Expression in the Colon

**Figure 6:**
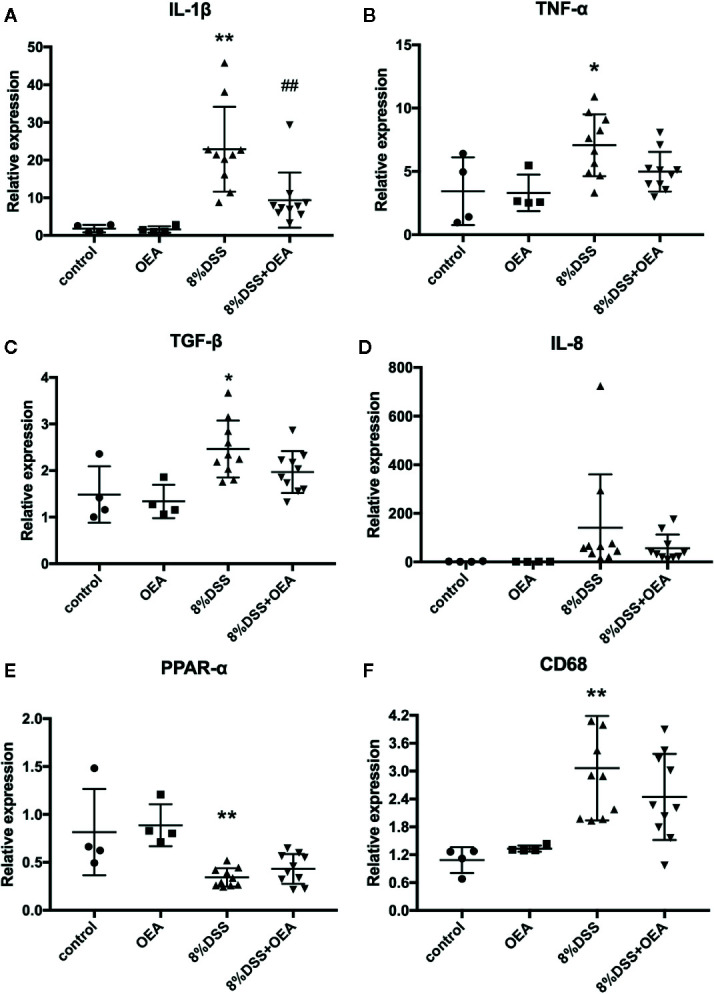
Gene expression analyses of the colon. qRT-PCR analyses of **(A)** IL-1β, **(B)** TNF-α, **(C)** TGF-β, **(D)** IL-8, **(E)** PPAR-α, and **(F)** CD68. The values are mean ± SD (n = 4, Control group; n = 4, OEA group; n = 10, DSS group and n = 10, DSS + OEA group). ^*^ *p <*0.05 versus Control group. ^**^ *p* < 0.01 versus Control group. ^##^ *p* < 0.01 versus DSS group.

We next examined the expression of inflammation-related genes in the colon. In the DSS group, mRNA expression levels of inflammatory cytokines, such as IL-1β, TNF-α, and transforming growth factor (TGF)-β, were significantly increased compared to the control group. In the DSS + OEA group, the expression levels of these cytokines tended to be reduced (**Figures 6B, C**) and the expression of IL-1β was significantly reduced compared to the DSS group (**Figure 6A**). The expression level of IL-8 tended to be increased in the DSS group compared to the control group and reduced in the DSS + OEA group compared to the DSS group, although not statistically significant (**Figure 6D**). The expression level of PPAR-α was significantly decreased by the DSS administration compared to the control group, and there was no significant difference in the expression level of PPAR-α between the DSS group and the DSS+OEA group (**Figure 6E**). The expression level of CD68 was significantly increased in the DSS group compared to the control group, and tented to be decreased in the DSS + OEA group compared to the DSS group (**Figure 6F**).

## Discussion

**Figure 7:**
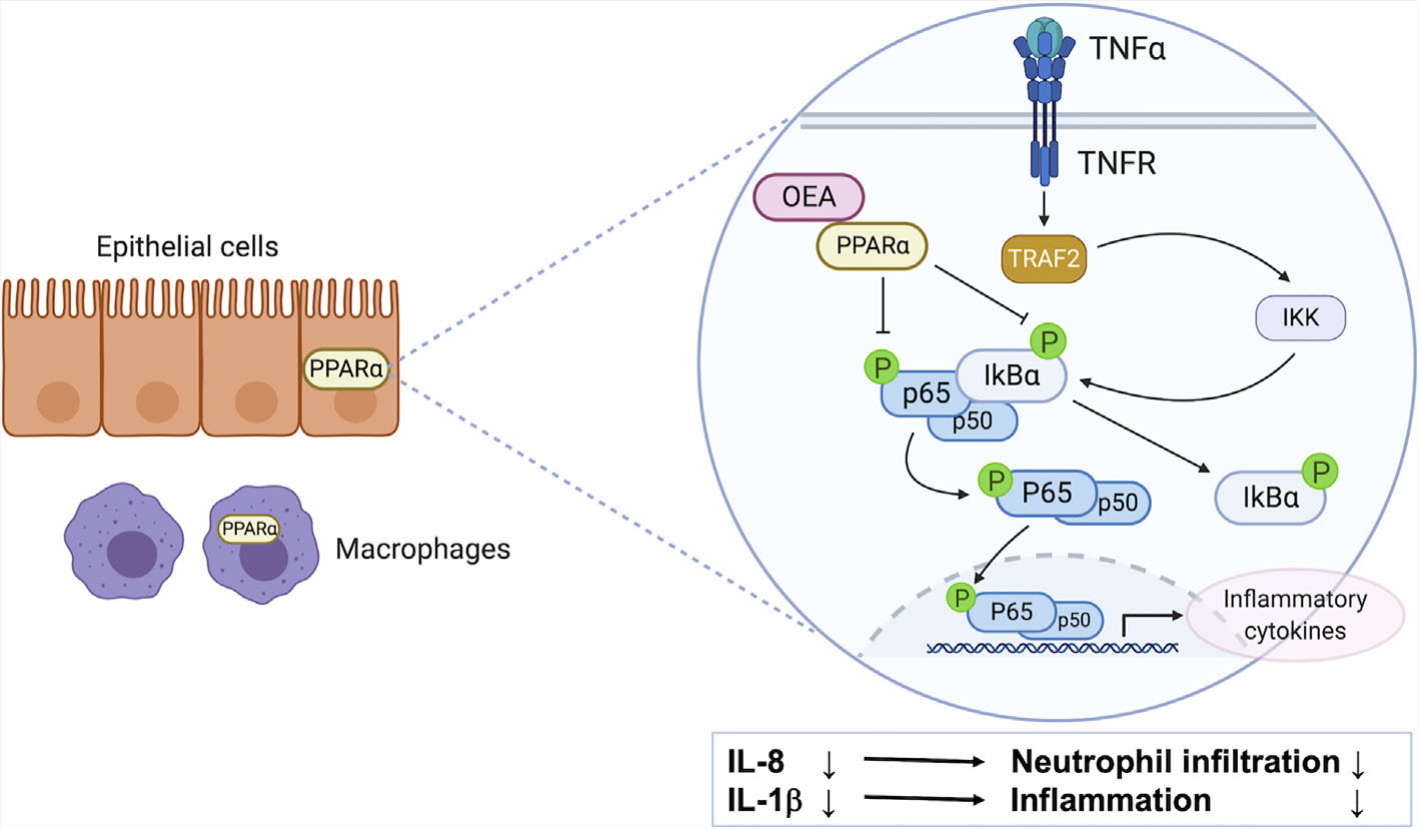
The possible mechanism of anti-inflammatory effect of OEA on colitis. PPAR-α is highly expressed in intestinal epithelial cells and macrophages. OEA inhibits phosphorylation of IκBα and p65, and suppresses the activation of the NF-κB signaling pathway, resulting in the reduction of the expression of IL-8 and IL-1β in epithelial cells. IL-8 reduction leads to suppression of neutrophil infiltration, and IL-1β reduction leads to suppression of inflammatory reaction in the colon.

In this study, we investigated the anti-inflammatory effects of OEA *in vitro* and *in vivo*, and investigated the underlying mechanisms through cell culture experiments. We found that (1) OEA suppressed the LPS-induced inflammatory reaction *in vitro*, (2) OEA suppressed the TNF-α-induced inflammatory reaction in cultured intestinal epithelial cells, and (3) OEA administration ameliorated DSS-induced colitis in rats (**Figure 7**).

The expression and activation of NF-κB was strongly induced in the inflamed gut of patients with IBD, and the amount of activated NF-κB was significantly correlated with the severity of intestinal inflammation (Atreya et al., 2008). OEA has been demonstrated to exert anti-inflammatory effects on LPS-induced monocytes (THP-1) by inhibiting the NF-κB and ERK1/2/AP-1/STAT3 pathways (Yang et al., 2016). In our study, OEA dose-dependently reduced the expression of TNF-α and inhibited phosphorylation of IκBα in LPS-induced 293/hTLR4A-MD2-CD14 cells. Furthermore, OEA reduced the expression of inflammatory cytokines and inhibited phosphorylation of IκBα and p65 in TNF-α-induced Caco-2 cells. These results suggest that OEA has anti-inflammatory effects, which could be caused by suppressing the activation of the NF-κB signaling pathway in intestinal epithelial cells.

OEA has been reported to function as an agonist for PPAR-α (Fu et al., 2005), TRPV1 (Ahern, 2003), and GPR119 (Lauffer et al., 2009). PPAR-α is highly expressed in intestinal cells (Kimura et al., 2013) and macrophages (Manoharan et al., 2016). It has been demonstrated that the PPAR-α agonist improves murine experimental colitis (Azuma et al., 2010) and deletion or disruption of the PPAR-α pathway alters the function of colonic macrophages (Manoharan et al., 2016). The role of TRPV-1 in the IBD model is controversial (Cseko et al., 2019), and the effects of the GPR119 agonist on the IBD model are unknown. In our study, a PPAR-α antagonist blocked the effect of OEA in reducing the expression of IL-8 *in vitro*. This result suggests that the anti-inflammatory effect of OEA in intestinal epithelial cells is mediated by PPAR-α signaling. It has been reported that the PPAR-α mRNA levels were decreased by LPS administration, and different concentrations of OEA upregulated the expression of PPAR-α in cultured monocytes (THP-1 cells, Yang et al., 2016), and it has been reported that administration of OEA significantly increased the mRNA expression levels of PPAR-α in nonalcoholic fatty liver of rats (Li et al., 2015). However, in the present study, the expression of PPAR-α in the colon was decreased by DSS administration, and the OEA administration did not ameliorate this reduction. This result may suggest that the OEA acts as an agonist for PPAR-α without upregulation of PPAR-α expression in the colon.

Macrophages produce pro-inflammatory cytokines in patients with IBD and can induce tissue damage (Steinbach and Plevy, 2014). In our *in vivo* study, macrophage infiltration was suppressed by the administration of OEA. In patients with UC, the extent of neutrophil infiltration correlates with the severity of disease (Wera et al., 2016), and IL-8 is mainly a neutrophil chemoattractant that induces the migration of neutrophils from peripheral blood into inflamed tissue (Lee et al., 2018). In the present study, OEA suppressed the expression of IL-8 in Caco-2 cells and neutrophil infiltration in the colon of rats. These results might suggest that OEA suppresses neutrophil infiltration in the colon by inhibiting IL-8 expression in intestinal epithelial cells.

It has been reported that administration of OEA reduces food intake (Fu and Gaetani, 2013). In several studies, food intake was significantly reduced by administration of DSS (Ye et al., 2009; Chaudhary et al., 2017). In our study, food intake in the OEA group and the DSS group was significantly reduced compared with that in the control group, as previously reported. However, there was no difference in food intake among the DSS group and the DSS+OEA group. This result might suggest that the anti-inflammatory effects of OEA nullify the reduction in food intake caused by DSS administration. In the present study, although significant reduction of food intake in the OEA group was observed compared to the control group, relative body weight was not affected. This may be explained by the influence of OEA on the reduction of spontaneous activity, as previously reported (Proulx et al., 2005).

The therapeutic effects of OEA had previously been reported in small animals with atherosclerosis (Fan et al., 2014), nonalcoholic fatty liver (Li et al., 2015), and neuroinflammation (Sayd et al., 2014). In a human clinical trial, the use of OEA for people with obesity improved inflammation and oxidative stress (Payahoo et al., 2018). OEA could have the potential for clinical use in various diseases in the future, and further clinical studies are needed.

This study has several limitations. (1) Although the PPAR-α experiment was conducted *in vitro*, experiments on other OEA receptors, such as TRPV-1 and GPR119, were not conducted. (2) OEA was administered only intraperitoneally, and oral administration was not conducted in the animal experiment. In many studies, OEA was administrated intraperitoneally and the dosage was typically 5–20 mg/kg. However, in several studies, OEA was administered orally and the dosage was 25–200 mg/kg (Nielsen et al., 2004; Oveisi et al., 2004; Thabuis et al., 2011). (3) OEA administration was started at day 0, before the colitis was developed. (4) Our experimental design was an acute severe colitis model rather than chronic colitis model.

In conclusion, OEA administration ameliorated DSS-induced colitis in rats, possibly by inhibiting the NF-κB signaling pathway through PPAR-α receptors in intestinal epithelial cells. OEA administration could represent a new therapeutic strategy for treating colitis.

## Data Availability Statement

The raw data supporting the conclusions of this article will be made available by the authors, without undue reservation.

## Ethics Statement

The animal study was reviewed and approved by the Animal Care Unit and Use Committees of Hokkaido University.

## Author Contributions

SOt contributed to all experiments, data analysis, and manuscript writing. SOh and NS contributed to conception and design, and final approval of the manuscript. MO, QF, KoY, KeY, and TK contributed to assembly of data and data analysis. All authors contributed to the article and approved the submitted version.

## Funding

This study was supported by a Grant-in-Aid for Scientific Research (C) from the Japan Society for the Promotion of Science (JSPS, 20K08347).

## Conflict of Interest

The authors declare that the research was conducted in the absence of any commercial or financial relationships that could be construed as a potential conflict of interest.

## Abbreviations

CD, Crohn’s disease; DAI, disease activity index; DMEM, Dulbecco’s modified Eagle’s medium; DMSO, dimethyl sulfoxide; DSS, dextran sulfate sodium; FBS, fetal bovine serum; GPR, G protein-coupled receptor; HE, hematoxylin and eosin; IBD, inflammatory bowel disease; Iκ inhibitor of kappa; IL, interleukin; LPS, lipopolysaccharide; MPO, myeloperoxidase; NF-κB, nuclear factor kappa B; OEA, oleoylethanolamide; PBS, phosphate-buffered saline; PPAR, peroxisome proliferator-activated receptor; qRT-PCR, quantitative reverse transcription-polymerase chain reaction; SD, standard deviation; SDS, sodium dodecyl sulfate; TGF, transforming growth factor; TLR, toll-like receptor; TNF, tumor necrosis factor; TRPV, transient receptor potential vanilloid; UC, ulcerative colitis.
